# Two divergent *Symbiodinium* genomes reveal conservation of a gene cluster for sunscreen biosynthesis and recently lost genes

**DOI:** 10.1186/s12864-018-4857-9

**Published:** 2018-06-14

**Authors:** Eiichi Shoguchi, Girish Beedessee, Ipputa Tada, Kanako Hisata, Takeshi Kawashima, Takeshi Takeuchi, Nana Arakaki, Manabu Fujie, Ryo Koyanagi, Michael C. Roy, Masanobu Kawachi, Michio Hidaka, Noriyuki Satoh, Chuya Shinzato

**Affiliations:** 1 Marine Genomics Unit, Okinawa Institute of Science and Technology Graduate University, Onna, Okinawa, 904-0495 Japan; 2 Present address: Department of Genetics, School of Life Science, The Graduate University for Advanced Studies, 1111, Yata, Mishima-shi, Shizuoka, 411-8540 Japan; 3 Present address: Center for Information Biology, National Institute of Genetics, Mishima, 411-8540 Japan; 4 DNA Sequencing Section, Okinawa Institute of Science and Technology Graduate University, Onna, Okinawa, 904-0495 Japan; 5 Instrumental Analysis Section, Okinawa Institute of Science and Technology Graduate University, Onna, Okinawa, 904-0495 Japan; 6 Center for Environmental Biology and Ecosystem Studies, National Institute for Environmental Studies, Tsukuba, 305-8506 Japan; 7 Department of Chemistry, Biology and Marine Science, University of the Ryukyus, Nishihara, Okinawa, 903-0213 Japan; 8 Atmosphere and Ocean Research Institute, The University of Tokyo, Kashiwanoha, Kashiwa, 277-8564 Japan

**Keywords:** Dinoflagellates, Evolutionary genomics, *Symbiodinium*, Mycosporine-like amino acids, Symbiosis

## Abstract

**Background:**

The marine dinoflagellate, *Symbiodinium*, is a well-known photosynthetic partner for coral and other diverse, non-photosynthetic hosts in subtropical and tropical shallows, where it comprises an essential component of marine ecosystems. Using molecular phylogenetics, the genus *Symbiodinium* has been classified into nine major clades, A-I, and one of the reported differences among phenotypes is their capacity to synthesize mycosporine-like amino acids (MAAs), which absorb UV radiation. However, the genetic basis for this difference in synthetic capacity is unknown. To understand genetics underlying *Symbiodinium* diversity, we report two draft genomes, one from clade A, presumed to have been the earliest branching clade, and the other from clade C, in the terminal branch.

**Results:**

The nuclear genome of *Symbiodinium* clade A (SymA) has more gene families than that of clade C, with larger numbers of organelle-related genes, including mitochondrial transcription terminal factor (mTERF) and Rubisco. While clade C (SymC) has fewer gene families, it displays specific expansions of repeat domain-containing genes, such as leucine-rich repeats (LRRs) and retrovirus-related dUTPases. Interestingly, the SymA genome encodes a gene cluster for MAA biosynthesis, potentially transferred from an endosymbiotic red alga (probably of bacterial origin), while SymC has completely lost these genes.

**Conclusions:**

Our analysis demonstrates that SymC appears to have evolved by losing gene families, such as the MAA biosynthesis gene cluster. In contrast to the conservation of genes related to photosynthetic ability, the terminal clade has suffered more gene family losses than other clades, suggesting a possible adaptation to symbiosis. Overall, this study implies that *Symbiodinium* ecology drives acquisition and loss of gene families.

**Electronic supplementary material:**

The online version of this article (10.1186/s12864-018-4857-9) contains supplementary material, which is available to authorized users.

## Background

Dinoflagellates are one of the major groups in the supergroup Alveolata, with an estimated ~ 2500 species [1]. They inhabit aquatic environments and nearly half are phototrophic [1]. Dinoflagellate evolution has been resolved using morphological and molecular phylogenetic analyses [2, 3]. Comparative analyses suggest that horizontal gene transfers are linked to major transitions in dinoflagellate evolution [4, 5].

Recent studies of dinoflagellates focused on their life styles in relation to marine environments [1, 6]. Dinoflagellates are the major group of red tide-producing microorganisms [7], specialized for toxin biosynthesis [8]. However, dinoflagellates of the genus *Symbiodinium* are renowned for their symbiotic relationships with reef-building corals [9, 10], which are foundational to marine ecosystem biodiversity [11–13].

**Fig. 1:**
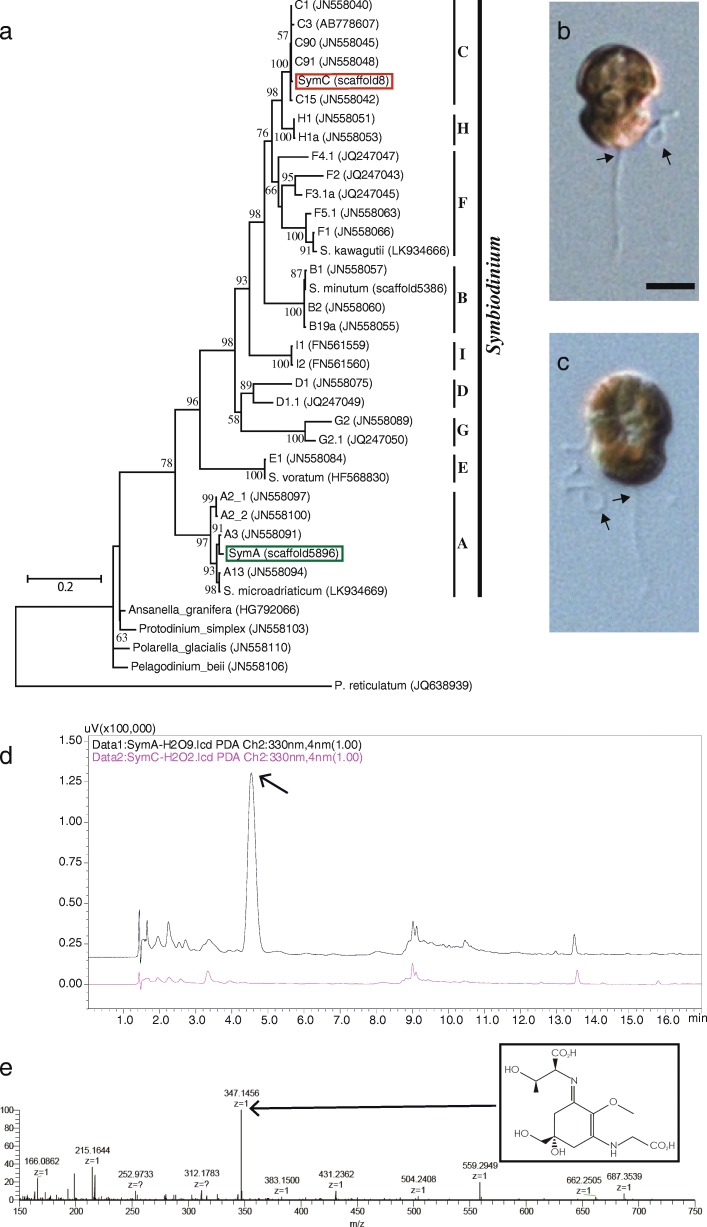
Phenotypic differences in the production of mycosporine-like amino acids of two divergent *Symbiodinium* species. **a.** Phylogenetic positions of the *Symbiodinium* species analyzed, SymA (green) and SymC (red). A phylogenetic tree was constructed using the Maximum-Likelihood method, based on 28S rDNA sequences [12]. The scale bar shows 0.2 changes per site. **b.** Zoospore of SymA. Scale bar, 5 μm. **c.** Zoospore of SymC. A short, transverse flagellum originating from the cingulum and a long longitudinal flagellum originating from the sulcus, are observed in zoospores (arrows). **d.** High-performance liquid chromatography (HPLC) comparison of aqueous extracts prepared from SymA (black) and SymC (pink) detected at 330 nm. The largest difference between SymA and SymC is seen in peaks with a retention time of ~ 4.5 min (arrow). The large peak in SymA is not detected in SymC. **e.** High-resolution mass spectrum of isolated Porphyra-334 (MH^+^ 347.1456, C_14_H_23_N_2_O_8_, Δ 0.74 mmu), showing the production of mycosporine-like amino acid (MAAs) by SymA (arrow). Inset shows the chemical formula of Porphyra-334

The extensive diversification of *Symbiodinium* has been well described [11–16]. Molecular phylogenetics has classified these dinoflagellates into nine major groups, A to I [17]. *Symbiodinium* strains are hosted by ciliates, foraminiferans, sponges, cnidarians, molluscs, and acoelomorphs [12, 18]. It is thought that clade A diverged first (the oldest) and that lineages C and H in the crown clade are the most recent (the youngest) (Fig. 1a). Clade A *Symbiodinium* may also form parasitic as well as mutualistic symbioses with other organisms [19–21]. The diversity and dominance of clade C in association with reef invertebrates has been reported in the Great Barrier Reef (GBR), Australia, and at Zamami Island, Okinawa, Japan [22].

Physiological work on *Symbiodinium* diversity has been reported using cultured *Symbiodinium* strains [23–25] and recent work clarifies differences in metabolite production among *Symbiodinium* clades [26]. One of the pioneering studies reported differences in mycosporine-like amino acid (MAA) production [23], but the genetic basis of this chemistry remains unknown in *Symbiodinium*. MAAs function as anti-oxidants and UV-absorbing molecules [27]. In a cultured dinoflagellate, *Gymnodinium sanguineum*, MAAs function as specific UV blockers to protect the dinoflagellate photosynthetic machinery [28]. Recently, four biosynthetic enzymes in a cyanobacterium were characterized using heterologous gene expression and an MAA biosynthetic gene cluster encoding those enzymes was characterized [29]. Further reports suggested that three enzymes involved in MAA biosynthesis, dimethyl 4-deoxygadusol (DDG), *O*-methyltransferase (*O*-MT), and ATP-grasp, are conserved in bacteria [30, 31] and the enzyme for the fourth biosynthetic step from the cluster is either a non-ribosomal peptide synthetase (NRPS) homolog or D-alanine (D-Ala) D-Ala ligase-like [30, 32]. Although these genes have also been found in eukaryotic genomes [33–37], the gene cluster has been identified only in bacterial [38] and red algal genomes [39]. One hypothesis is that the capacity for MAA production in *Symbiodinium* may be essential for symbioses involving hosts that cannot produce MAAs [27, 40].

Draft genomes have been published for three *Symbiodinium* taxa [4, 41, 42]. The most recent report [42] focused on diversification of transmembrane transporter genes. Comparative analysis also described the importance of duplicated genes as an evolutionary mechanism, underscoring the importance of lineage-specific expansions for symbiotic lifestyles, especially for genes encoding ion transporters. Comparative transcriptomic analyses have identified possible lineage- or clade-specific gene families [43]. While genome evolution of parasitic apicomplexans has been extensively studied, genomes of symbiotic dinoflagellates are still comparatively little known. Therefore, comparative genomic studies of diverse *Symbiodinium* species are essential to better understand *Symbiodinium* diversity. To clarify the genetic basis for different physiological phenotypes, we decoded the genomes of culturable clade A and clade C *Symbiodinium* and performed comparative analyses.

## Results

### Genome assembly and physiological characters in divergent *Symbiodinium* taxa

To obtain *Symbiodinium* genome sequences from early- and late-branching lineages, two strains (clade A, Y106 and clade C, Y103) were selected for genome sequencing (Fig. 1b and c). A molecular phylogenetic tree (Fig. 1a) from the recent analysis by Pochon et al. [12] and ITS sequences showed that Y106 belongs to clade A3 (SymA). In contrast, the subclade of Y103 was not clear from our phylogenetic analysis (SymC), although its ITS suggested that it is similar to clade C92 [44].

Physiological characterization by mass spectrometry confirmed that SymA3 produces an MAA, Porphyra-334 (Fig. 1d and e), which has an *m/z* of 347.1456 Da. On the other hand, MAAs were not detected in SymC (Fig. 1d) or in *S. minutum* (shown here as SymB) of clade B1 (data not shown). These results are comparable to previously reported differences at the clade level [23].

Using genomic DNA and mRNA from cloned cells, we obtained sequence data using Illumina GAIIx and Hiseq sequencers (Additional file 1: Tables S1 and S2). Approximately ~ 200 Gbp of genomic sequences were used for each assembly (Additional file 1: Table S1). In our previous sequencing of the *S. minutum* (SymB) genome, sequence data amounted to ~ 56 Gbp from Roche 454 and Illumina GAIIx platforms [4]. Thus, the amount of raw data in this study was much greater than that of the previous study. The assembly was performed as described previously, with several modifications [4]. We produced two assembled genomes suitable for gene analyses (Additional file 1: Table S3), as in our previous report [4]. Scaffolds totaled ~ 767 Mb and ~ 705 Mb for SymA and SymC, respectively (Additional file 1: Table S3). RNAseq data were derived from *Symbiodinium* cultured under standard conditions, as described previously [4], or under dark conditions (Additional file 1: Table S2). Data were assembled into 76,628 unique cDNAs in SymA and 68,876 in SymC (Additional file 1: Table S4). Gene predictions yielded 69,018 and 65,832 protein-coding models, respectively (Additional file 1: Table S3). The following genome browser provides access to the assembled data and predicted genes: http://marinegenomics.oist.jp/gallery/ [45]. Using TopHat with default parameters [46], RNAseq reads were mapped by library (Additional file 1: Table S2) onto the draft genomes and information for read counts is available on the browser (Additional file 2: Figure S1). For SymA, 67.5% of the gene models were supported by RNAseq data and 62.5% for SymC. A characteristic feature of gene structures in SymC was a higher frequency of genes lacking introns (~ 19.7%) (Additional file 1: Table S3). The GC contents of the assembled SymA and SymC genomes were 50 and 43%, respectively (Additional file 1: Table S3). Unidirectional arrangements of genes and three major types (GT/GC/GA) of the first two nucleotides of introns [4] were found in the genomes of SymA and SymC (Additional file 1: Table S3).

### Gene content of each *Symbiodinium* genome

**Fig. 2:**
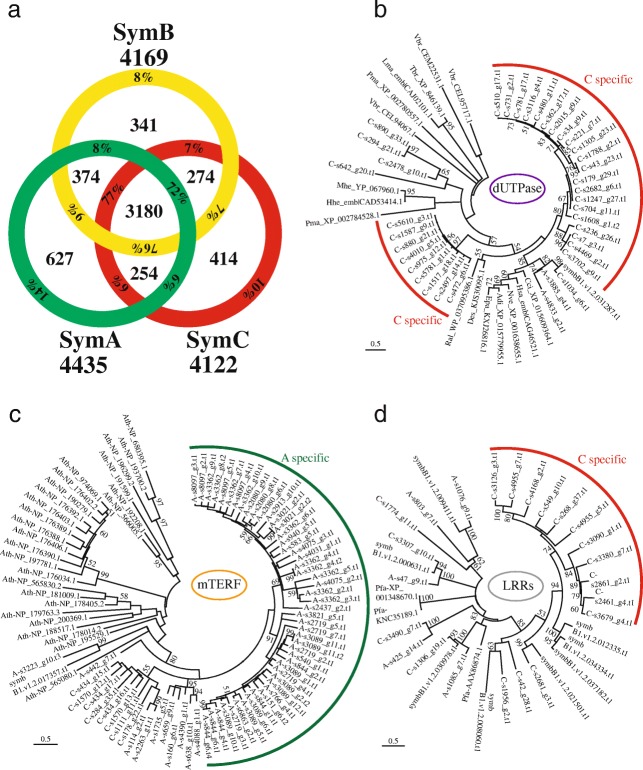
Comparisons and expansions of gene families in *Symbiodinium* lineages. **a.** Venn diagram comparing Pfam domains among three divergent *Symbiodinium* genomes. Total numbers of domain types found in each genome are shown outside the circle. Percentages indicate the ratio of the inside number to the total number. **b-d.** Molecular phylogenies of dUTPase (b), mitochondrial transcription termination (mTERF) proteins (c), and LRR domain-containing proteins (d), respectively. Scale bars show 0.5 changes per site. The arcs show possible lineage specific expansions in SymC (red) and SymA (green), respectively. A, SymA. B, symbB1. C, SymC. Adi, *Acropora digitifera*. Ath, *Arabidopsis thalina*. Cci, *Cephus cinctus*. Des, *Desulfatitalea sp.* Epa, *Exaiptasia pallida*. Hhe, *Human herpesvirus*. Hsa, *Homo sapiens*. Lma, *Leishmania major*. Mhe, *Macacine herpesvirus*. Nve, *Nematostella vectensis*. Pma, *Perkinsus marinus*. Ral, *Rhizobium alamii*. Tbr, *Trypanosoma brucei*. Vbr, *Vitrella brassicaformis*

Both genomes were predicted to contain more than 65,000 genes (69,018 for SymA and 65,832 for SymC) (Additional file 1: Table S3). These numbers are larger than those of previously reported *Symbiodinium* genomes (41,925 for *S. minutum*, 36,850 for *S. kawagutii*, and 49,109 for *S. microadriaticum*) [4, 41, 42], although they fall within the estimated range of 38,000–87,000 [47]. To clarify which gene families are conserved or expanded in each lineage, we annotated predicted proteins using a pfam domain search (http://marinegenomics.oist.jp/gallery/) and compared the proteins with genes of *S. minutum*. We found 4435 domain classes for 26,261 SymA genes, 4169 for 21,107 SymB genes, and 4122 for 23,808 from SymC (Fig. 2a).

Next, we compared gene numbers within gene families in each genome. Lineage-specific gene family expansions were defined as Pfam domain groups with multiple copies in *Symbiodinium,* in which gene numbers were significantly greater in one genome compared to the other two. The 30 most expanded gene families are summarized for SymA (Additional file 1: Table S5) and SymC (Additional file 1: Table S6), respectively. Our analyses indicate that the majority of the top 30 Pfam domains in SymA correspond to those reported previously [42]. These include reverse transcriptase (RVT), regulator of chromosome condensation (RCC1), and endonuclease (Additional file 1: Table S5). In the SymC genome, gene families for RVT, DNA methylase, integrase, and zf-CCHC are expanded. Thus, comparisons of gene numbers with Pfam domains showed many copies of reverse transcriptase in SymA and SymC. Special expansions in the genome of the late-branching group were predicted in gene families with DNA methylase or zf-CCHC domains. Similar observations have been reported in the *Symbiodinium kawagutii* genome [41]. It is possible that DNA methylation is related to endogenous retroviral expression [48]. zf-CCHC domains have been found in retrovirus GAG proteins [49]. These larger gene numbers in SymA and SymC (Additional file 1: Table S3) seem to be partly related to the richness of enzyme genes in many retroviruses.

To confirm the relationship between lineage-specific expansions and potential retrogenes, we constructed a molecular phylogenetic tree of UTPase proteins from *Symbiodinium* genomes (Fig. 2b). dUTPases prevent the misincorporation of uracil into DNA, and these enzymes have recently been suggested to regulate host interactions [50]. The two *Symbiodinium* genomes (SymA and SymB) encode one or two eukaryotic dUTPases per genome (Fig. 2b). On the other hand, the dUTPases in SymC are expanded and some of them had RVT domains. In addition, many of them are intronless, suggesting that gene expansion in SymC is due to integration of processed cDNAs [51].

### Genes of endosymbiotic origin are conserved in the early-branching genome

When proteins containing transposable elements or retrovirus-related Pfam domains were removed from the calculation, it became apparent that organelle-related genes (mitochondrial or plastid proteins) have been expanded in SymA (Additional file 1: Table S5). In particular, the gene expansion for mitochondrial transcription termination factor family protein (mTERF) was found in the early-diverging genome (Fig. 2c). *mTERF* genes have been identified as putative organellar transcription factors [52, 53]. Land plants have the highest number of *mTERF* genes (~ 30 members), which are targeted to plastids and mitochondria [54]. The mammalian mTERF family (four genes) is important in mitochondrial gene expression. In addition, gene numbers for Peridinin-chlorophyll A binding protein (PCP), chlorophyll A-B binding proteins, and Rubiscos were more numerous than those of other *Symbiodinium*. Expansion of chlorophyll a-binding proteins has also been reported in *Symbiodinium minutum* [55]. Several genes for Rubisco are tandemly aligned in the SymA genome, consistent with a previous report in the dinoflagellate, *Prorocentrum minimum* [56]. Differences in plastid physiological responses to heat stress were analyzed in SymA and SymB [57] and may be due to the expanded plastid-related proteins. In a future study, the relationship between stress and expansion of organelle-related genes will be determined, although gene functions in organelle genomes might also be important to understand differences in sensitivity to heat and light stress [58, 59].

### Expansions of repeat domain-containing genes in the late-branching genome

There were fewer gene families in the SymC genome than in SymA or SymB. On the other hand, genes for repeated domains are expanded, including leucine-rich repeats (LRR), FNIP (initial “FNIP” amino acids) repeats, and tetratrico peptide repeats (TPR) (Additional file 1: Table S6). Those domains are involved in protein-protein interactions [60, 61]. Therefore, these expansions may be similar to those of apicomplexans [62]. To characterize expanded LRR-containing proteins, we performed molecular phylogenetic analyses. Most of the expansion in SymC pertained to one subfamily similar to FNIP repeats, which has also been expanded in the *Dictyostelium discoideum* genome [63]. Other proteins with expanded LRRs were similar to those of sds22 and PfLRR1, which relate to cell cycle regulation [64]. Molecular phylogeny showed that lineage-specific expansions occurred in both SymB and SymC (Fig. 2d), suggesting that numbers of genes for protein-protein interactions are expanded in the late-branching genome.

### Gene cluster for mycosporine-like amino acid (MAA) biosynthesis in *Symbiodinium*

**Fig. 3:**
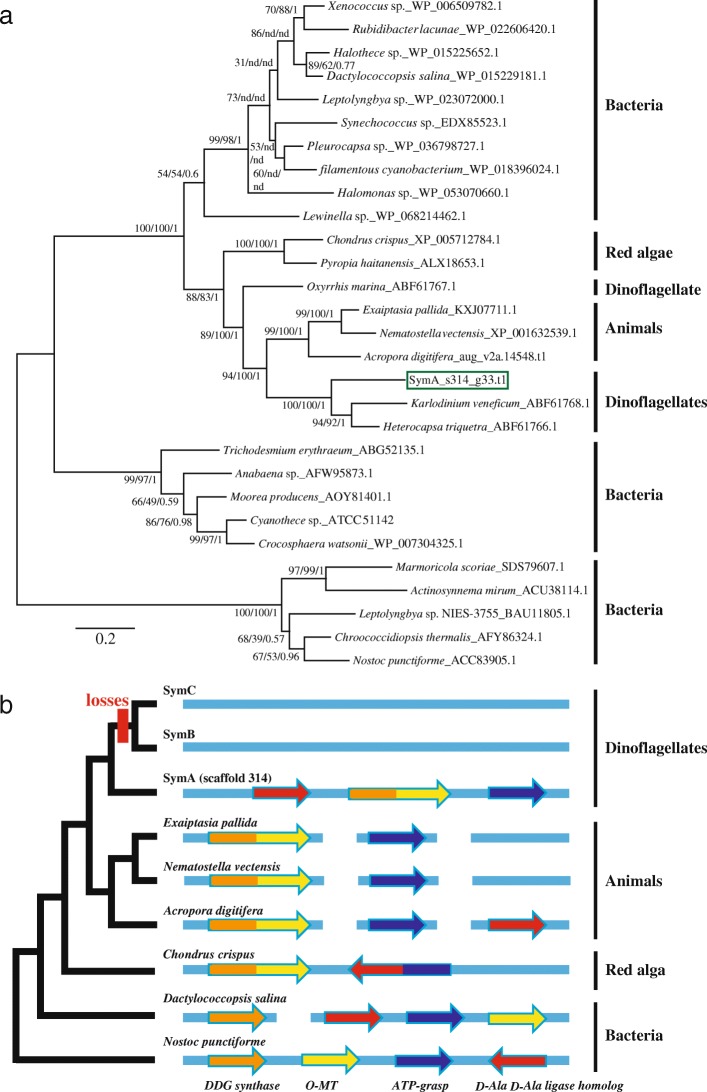
The nuclear genome of the earliest-branching *Symbiodinium* (SymA) harbors a gene cluster to produce MAAs. **a.** Molecular phylogenetic analysis of dimethyl 4-deoxygadusol (DDG) synthase by maximum likelihood. Numbers denote bootstrap values (WAG+G model and RAxML) and posterior probabilities (MrBayes). nd: “not determined” by a different topology. DDG genes of *Symbiodinium* are clustered with other dinoflagellate proteins from *Karlodinium veneficum* and *Heterocapsa triquetra*. In addition, dinoflagellate proteins are phylogenetically close to those of anthozoans. **b.** Potential evolutionary relationships between MAA gene clusters in cyanobacteria and those of eukaryotes with DDG synthase are shown. Deep evolutionary conservation of the bacterial-like gene cluster is suggested in SymA. On the other hand, genes within the cluster have been lost in the crown lineage (red box). The topology of the tree is based on phylogenetic analysis of DDG synthase

To determine the genetic basis for the difference in MAA production (Fig. 1d and e), we surveyed the decoded *Symbiodinium* genomes. MAA biosynthetic genes have not been found in the genome of *Symbiodinium kawagutii* [41], but they have been identified in the host genomes of cnidarian anthozoans [35, 36]. We found genes for MAA biosynthesis [29, 30] in the SymA genome. In addition, preliminary RNAseq analysis indicated that expression levels of those genes were similar between light and dark conditions (Additional file 2: Figure S1). Unexpectedly, the gene cluster corresponds to that of bacteria, although the gene arrangement of *D-Ala D-Ala ligase* differs from the bacterial arrangement (Fig. 3). We constructed four phylogenetic trees incorporating the genes in this cluster (Fig. 3a, Additional file 2: Figures S2-S4). Dinoflagellate DDG synthases clustered with those of anthozoans (Fig. 3a), while *O*-methyltransferases and D-Ala D-Ala ligases are shared with those of bacteria (Additional file 2: Figures S2 and S4). The phylogenetic relationship of ATP-grasp is unclear (Additional file 2: Figures S3). This complicated result suggests that those genes have been lost in eukaryotes or have been transferred several times. It has been suggested that the fusion gene (3-dehydroquinate synthase+*O*-methyltransferase) came from cyanobacteria [33] or via secondary endosymbiosis [65]. Our analysis implies that these genes were likely acquired in gene transfers via secondary endosymbiosis (Fig. 3a).

## Discussion

Our comparative analysis identified genomic characters of SymA and SymC, both of which were originally isolated from bivalve molluscs. The higher GC content of the SymA genome was similar to that reported in *S. microadriaticum* [42], suggesting that this may be an attribute of the earliest-branching lineage. Comparisons of gene families suggest that the late-branching lineage has lost more gene families than early-branching lineages, or that the early-branching lineages have acquired more gene families than the late-branching lineage. In other words, in SymC, there are fewer gene families, even though total gene numbers are expanded in late-branching *Symbiodinium*.

Finally, we found that the genome of SymA in the early-branching clade encoded a gene cluster for MAA biosynthesis. As this gene cluster is conserved, the transfer of large DNA segments probably occurred at an early stage of endosymbiosis. However, we cannot exclude the possibility that the cluster formed in the *Symbiodinium* lineage. Our survey shows that the three genes for MAA biosynthesis are found in *S. microadriaticum* and their genomic locations are dispersed on three scaffolds, 22, 397 and 882 [66] (http://smic.reefgenomics.org). Differences between the two genomes of clade A *Symbiodinium* also support reports of diversity in this early diverging lineage [20, 21]. Although it is suggested that adaptation to shallow-water environments may have been maintained in clade A *Symbiodinium* [67], previous reports for 54 species of symbiotic cnidarians have shown that highly variable MAA concentrations are not depth-dependent [68]. On the other hand, genes in this cluster were not found in the SymC genome. Although we surveyed raw data from publicly available *Symbiodinium* genomes by BLAST search, orthologs of the fusion gene (3-dehydroquinate synthase+*O*-methyltransferase) were not found in SymB, *S. kawagutii* or SymC (data not shown). Since SymB and *S. kawagutii* (clade F) genomes also lack these genes [4, 41], it is likely that gene losses occurred in the common ancestor of the crown lineages.

Loss of the capacity to produce UV-absorbing molecules may have been compensated by expansion of other genes for UV stress. We surveyed possible enzymes for repairing UV-damaged DNA [69] because cryptochromes/photolyases in dinoflagellates have not been surveyed in detail. Molecular phylogenetic analyses revealed no large differences in such gene families. Genomes of *Symbiodinium* encode three groups of cryptochromes/photolyases [69] (Additional file 2: Figure S5). Therefore, it is difficult to conclude that there is any relationship between acquisitions of repair genes and losses of MAA biosynthetic genes. On the other hand, diverse MAAs have been detected in coral tissues [27, 70] and in shallow-water bivalves [40], so adaptation to UV radiation may depend largely on symbioses with MAA-producing or -using hosts [40, 71]. For example, a report about *Symbiodinium* evolution and bivalve symbiosis suggests that the *Symbiodinium* in the bivalve, *Fragum*, might be a shade-loving alga [72]. SymC, which was originally isolated from *Fragum*, had no MAA biosynthetic gene cluster, so our analysis supports that suggestion [72].

## Conclusions

Gene expansions in *Symbiodinium* have occurred both by tandem duplication and integration of processed cDNA, possibly transposon-mediated. Comparative analyses indicate that expanded genes in the early-branching lineage include organelle-related genes. The crown lineage retains fewer gene families, but has acquired repeat-domain genes for protein-protein interactions, resembling massive gene losses and extracellular protein expansions in apicomplexans [62]. Finally, our decoded genomes show that the MAA gene cluster of secondary endosymbiotic origin, which is present in some dinoflagellate genomes, has been lost in the crown lineage of *Symbiodinum*. Taken together, these studies suggest that gene losses and expansions of genes transferred via secondary endosymbiosis drive *Symbiodinium* evolution.

## Methods

### Biological materials

Two dinoflagellates, *Symbiodinium* spp. clade A (SymA) and clade C (SymC) were cultured to produce genomic DNA and mRNA for sequencing. SymA and SymC are harbored by the cardiid clams, *Tridacna crocea* and *Fragum* sp., respectively, obtained in Okinawa, Japan. In regard to host habitats, *T. crocea* is epifaunal and *Fragum* is infaunal [72]. In the 1980s, isolations of *Symbiodinium* cells were performed by Prof. Terufumi Yamasu at the University of the Ryukyus using sterilized seawater and micropipettes [73]. The cultured *Symbiodinium* have been maintained since then in the laboratory of Prof. Michio Hidaka, at the University of the Ryukyus. SymA and SymC were designated as strains “Y106” and “Y103,” respectively. By manually isolating single cells under a microscope using a glass micropipette, each isoclonal line was established at the Marine Genomics Unit of Okinawa Institute of Science and Technology Graduate University in 2009. Repetitive subculture in 250-mL flasks has continued for 8 years, as previously described [4]. Using an incubator (CLE-303, TOMY), all cultures were maintained at 25 °C on a 12 h-light/12-dark cycle at about 20 μmol.m^− 2^ s^− 1^ illumination with white fluorescent lamps. The culture solution was artificial seawater containing 1× Guillard’s (F/2) marine-water enrichment solution (Sigma-Aldrich), plus three antibiotics, ampicillin (100 μg/mL), kanamycin (50 μg/mL), and streptomycin (50 μg/mL). Although culturing difficulties for some clade C *Symbiodinium* have been reported [74], the same culturing conditions have resulted in similar growth rates for SymA, SymC, and *S. minutum* (SymB).

### Genome sequencing and assembly

DNA obtained from clonal cultures (25 °C) of SymA and SymC was used for Illumina library construction (Additional file 1: Table S1), as described previously. Libraries were sequenced using the Illumina Genome Analyzer IIx (GAIIx) and Hiseq (Additional file 1: Table S1). Paired-end reads were assembled de novo with IDBA_UD (ver. 1.1.0) [75], and subsequent scaffolding was performed with SSPACE (ver. 3.0) [76] using Illumina mate-pair information. Gaps inside scaffolds were closed with Illumina paired-end data using Gapcloser [77]. As described previously [4], sequences that aligned to another sequence by more than 70% using BLASTN (1e^− 100^) were removed from the assembly. Scaffolds > 1 kb were added in version 1.0 of the genome assembly.

### Transcriptome sequencing and assembly

Cells cultured at 25 °C were aliquoted and freshly cultured under three types of conditions, 25 °C on 12 h-light/12-dark (Control), 31 °C on 12-light/12-dark (heat-stressed), and 25 °C under 24-dark (dark condition) (Additional file 1: Table S2). After 48 h, cells were collected and frozen for RNA extraction, as done previously [4]. RNAseq library preparation followed manufacturer protocols. RNA sequencing was performed using the GAIIx platform. De novo transcriptome assembly was performed using Trinity software [78].

### Gene prediction and annotation

A set of gene model predictions (Gene Model ver. 1) was generated mainly with AUGUSTUS [4], and a genome browser has been established using the Generic Genome Browser (JBrowser) [79]. Annotation and identification of *Symbiodinium* genes were performed using three methods or combinations of methods: reciprocal BLAST analyses, screening of gene models against the Pfam database [80] at an E-value cutoff of 0.001, and phylogenetic analyses. Gene annotations are available at the genome browser site (http://marinegenomics.oist.jp/gallery/). Scaffold 1 of both SymA and SymC manifested similarities to a bacterial genome, which was identified by genome sequencing of *Symbiodinium minutum* [4], but which was not included for gene annotation. Expansions of gene families were predicted by chi-square values from comparisons of gene numbers with Pfam domains.

### Molecular phylogenetic analysis

Maximum likelihood (ML) phylogenetic trees were constructed using MEGA 5.2, as previously described [81]. ML phylogenetic analysis of the DDG synthase family was also carried out using RaxML with 1000 bootstraps and using the GAMMA and Le-Gasquel model of rate heterogeneity [82]. Bayesian inference was conducted with MrBayes v.3.2 [83] using the same replacement model and run for 4 million generations and four chains until the posterior probability approached 0.01. Statistics and trees were summarized using a burn-in of 25% of the data. Phylogenetic trees were visualized using Figtree (http://tree.bio.ed.ac.uk/software/figtree/) and edited with Treegraph 2 [84].

### MAA extraction from *Symbiodinium*

*Symbiodinium* cells were cultured at 25 °C for 1 mo on a 12 h-light/12-dark cycle at about 20 μmol.m^− 2^ s^− 1^ illumination, as described in the section, Biological materials, and cells were not exposed to UV. Biomass was collected by centrifugation and extracted with methanol (10 mL × 2) at room temperature. Methanol (10 mL) was added to the biomass (0.4–0.6 g, wet weight) followed by vortexing (1 min), sonication (10 min), and centrifugation (7000 g, 10 min, 10 °C) to yield a methanol extract. The resulting clear solution was transferred to a new tube and stored at − 30 °C. Additional methanol was added to the residue, vortexed, and kept overnight at room temperature. After centrifugation, the second methanol extract was decanted, pooled with the first extract, and dried in a vacuum concentrator (40 °C), and the crude extract was stored at − 30 °C before HPLC analysis and purification. The dried methanol extract was suspended in TFA (0.2%, 1 mL) followed by vortexing (1 min), sonication (10 min), and centrifugation (7000 g, 10 min, 10 °C) to give a clear aqueous solution, which was collected and analyzed by HPLC and LC-MS.

### MAA analysis by high performance liquid chromatography (HPLC)

HPLC was run on a Nexera (LC-10 AD, Shimadzu) equipped with an autosampler (SIL-30 AC), a column oven (CTO-20 AC), and diode-array detector (SPD-M20A). An ODS column (150 × 2.1 mm, 5 μm, Hypersil Gold, Thermo) was used for MAA analysis and an ODS column (250 × 4.6 mm, 5 μm, Cosmosil) was used for purification. A 16-min gradient was used (A/B 100/0 for 0.0–5.0 min, 100/0 to 85/15 for 5.0–10.0 min, followed by washing 5/95 for 10.0–13.0 min and equilibration 100/0 for 13.0–16.0 min. Solvents (A) Milli Q water and (B) acetonitrile, both containing 0.1% TFA) were used for separation of compounds. A 15 μL sample was injected into the column using the auto-sampler and MAAs were detected at λ330 nm. A constant flow rate 300 μL/min was used and the column was kept at 25 °C.

### MAA crude extracts purification by HPLC

The aqueous MAA extract from *Symbiodinium* was dried and redissolved in 0.2% TFA (300 μL) and injected into the preparative ODS column (250 × 4.6 mm, 5 μm, Cosmosil) using the above HPLC and the target peak (retention time, 8.0 min) was collected. The purified component showed homogeneity in HPLC analysis and was identified as porphyrin-334 by high-resolution mass spectrometry.

### Identification of MAAs from *Symbiodinium* by NanoLC-mass spectrometry (NanoLC-MS)

As described previously [8], a Thermo Scientific hybrid (LTQ Orbitrap) mass spectrometer was used for MS data collection. The mass spectrometer was equipped with an HPLC (Paradigm MS4, Michrom Bioresources Inc.), an auto-sampler (HTC PAL, CTC Analytics), and a nanoelectrospray ion source (NSI). The high-resolution MS spectrum was acquired at 60,000 resolution in FTMS mode (Orbitrap), full mass range *m/z* 150–500 Da, with capillary temperature (200 °C) and spray voltage (1.9 kV), in positive ion mode. Crude extracts and purified MAA were separated on a capillary ODS column (50 × 0.15 mm, 3-μm, C_18_, Vydac). A 15-min gradient was employed (100% A for 0.0–10.0 min, 100 to 50% A from 10.1 to 12.0 min, hold at 50% A until 15.0 min, equilibration at 100% A for 15.0 to 18.0 min, where solvent A was water-acetonitrile 98:2 and solvent B was water-acetonitrile 2:98, both containing 0.1% formic acid. The flow rate was 2.0 μL/min and a 2.0 μL sample loop was used for MAA separation.

## Additional files

Additional file 1: **Table S1.** Summary of Illumina data used for assembling *Symbiodinium* genomes. **Table S2.** Summary of Illumina data used for assembling *Symbiodinium* transcriptomes. **Table S3.** Genomic compositions of three genomes of the genus *Symbiodinium*. **Table S4.** Summary of assembled transcriptome contigs. **Table S5.** Expanded genes having Pfam domains in SymA. **Table S6.** Expanded genes having Pfam domains in SymC. (DOCX 119 kb)

Additional file 2: **Figure S1.** A screen shot of scaffold 314 on the SymA genome browser, which is accessible via http://marinegenomics.oist.jp/gallery/, and RNAseq read counts showing the expressions of MAA biosynthetic genes. **Figure S2.** A molecular phylogenetic tree of *O*-methyltransferase. **Figure S3.** A molecular phylogenetic tree of ATP-grasp family proteins. **Figure S4.** A molecular phylogenetic tree of D-Ala D-Ala ligase family proteins. **Figure S5.** A molecular phylogenetic tree of cryptochromes/photolyases. (PDF 651 kb)
