# Supplementary material for: Two divergent Symbiodinium genomes reveal conservation of a gene cluster for sunscreen biosynthesis and recently lost genes
Source: BMC Genomics. 2018 Jun 14;19:458. doi: 10.1186/s12864-018-4857-9 (PMC6001144; doi:10.1186/s12864-018-4857-9)
Supplement: Supplementary file 1 — Table S1. Summary of Illumina data used for assembling Symbiodinium genomes. Table S2. Summary of Illumina data used for assembling Symbiodinium transcriptomes. Table S3. Genomic compositions of three genomes of the genus Symbiodinium. Table S4. Summary of assembled transcriptome contigs. Table S5. Expanded genes having Pfam domains in SymA. Table S6. Expanded genes having Pfam domains in SymC. (DOCX 119 kb) [file 12864_2018_4857_MOESM1_ESM.docx]

**Table S1. Summary of Illumina data used for assembling *Symbiodinium* genomes**

| Strain name of *Symbiodinium* spp. | Methods, library* | Total sequences (Gb) | Number of reads (million) | Read length (bp) | Accession number |
| --- | --- | --- | --- | --- | --- |
| clade A Y106 | Illumina GAIIx, 300-500 bp PE | 31.34 | 310.28 | 2 x 101 | DRR079237 |
| clade A Y106 | Illumina GAIIx, 600-800 bp PE | 31.92 | 316.06 | 2 x 101 | DRR079237 |
| clade A Y106 | Illumina GAIIx, 300-500 bp PE | 32.08 | 317.61 | 2 x 101 | DRR079237 |
| clade A Y106 | Illumina GAIIx, 600-800 bp PE | 31.85 | 315.33 | 2 x 101 | DRR079237 |
| clade A Y106 | Illumina GAIIx, 300-500 bp PE | 58.21 | 385.51 | 2 x 150 | DRR119200 |
| clade A Y106 | Illumina GAIIx, 500-700 bp PE | 47.28 | 313.10 | 2 x 150 | DRR119201 |
| clade C Y103 | Illumina Hiseq, 200-400 bp PE | 125.32 | 1240.80 | 2 x 150 | DRR088460 |
| clade C Y103 | Illumina Hiseq, 400-600 bp PE | 79.27 | 784.87 | 2 x 150 | DRR088461 |
| clade A Y106 | Illumina Hiseq, 1.5-4.0 kbp MP | 0.82 | 6.87 | 2 x 150 | DRR079238 |
| clade A Y106 | Illumina Hiseq, 4.0-6.0 kbp MP | 0.65 | 5.40 | 2 x 150 | DRR079239 |
| clade A Y106 | Illumina Hiseq, 6.0-8.0 kbp MP | 0.36 | 2.95 | 2 x 150 | DRR079240 |
| clade A Y106 | Illumina Hiseq, 8.0-12.0 kbp MP | 0.24 | 2.01 | 2 x 150 | DRR079241 |
| clade C Y103 | Illumina Hiseq, 1.5-4.0 kbp MP | 2.85 | 25.09 | 2 x 150 | DRR088462 |
| clade C Y103 | Illumina Hiseq, 4.0-6.0 kbp MP | 3.00 | 26.28 | 2 x 150 | DRR088463 |
| clade C Y103 | Illumina Hiseq, 6.0-8.0 kbp MP | 1.94 | 16.89 | 2 x 150 | DRR088464 |
| clade C Y103 | Illumina Hiseq, 8.0-12.0 kbp MP | 2.34 | 20.51 | 2 x 150 | DRR088465 |
| *PE: Paired End; MP: Mate Pair | |  |  |  |  |

**Table S2. Summary of Illumina data used for assembling *Symbiodinium* transcriptomes**

| Strain name of *Symbiodinium* spp. | library | Total sequences (Gb) | Number of reads (million) | Read length (bp) | Accession number |
| --- | --- | --- | --- | --- | --- |
| clade A Y106 | Control: 0h | 8.4 | 83.4 | 2 x 101 | DRR079242 |
| clade A Y106 | Control: 48h | 7.4 | 73.7 | 2 x 101 | DRR079243 |
| clade A Y106 | Dark condition: 48h | 8.5 | 84.4 | 2 x 101 | DRR079244 |
| clade A Y106 | Heat stress: 48h | 8.4 | 83.2 | 2 x 101 | DRR079245 |
| clade A Y106 | Heat stress in dark condition: 48h | 7.7 | 76.9 | 2 x 101 | DRR079246 |
| clade C Y103 | Control: 0h | 7.6 | 75.9 | 2 x 101 | DRR088466 |
| clade C Y103 | Control: 48h | 7.6 | 75.4 | 2 x 101 | DRR088467 |
| clade C Y103 | Dark condition: 48h | 8.0 | 79.5 | 2 x 101 | DRR088468 |
| clade C Y103 | Heat stress: 48h | 6.7 | 66.7 | 2 x 101 | DRR088469 |
| clade C Y103 | Heat stress in dark condition: 48h | 8.7 | 86.9 | 2 x 101 | DRR088470 |

**Table S3. Genomic compositions of three genomes of the genus *Symbiodinium***

|  |  |  |  |  |
| --- | --- | --- | --- | --- |
|  |  | *Symbiodinium minutum* (SymB)* | *Symbiodinium* sp. clade A (SymA) | *Symbiodinium* sp. clade C (SymC) |
|  |  |  |  |  |
|  | A total assembled length of assembly (bp) | 615,520,517 | 766,659,703 | 704,779,698 |
|  | scaffold N50 | 126.2k | 133.4k | 248.9k |
|  | G+C content (%) | 43.6 | 49.9 | 43.0 |
| Genes | No. of genes | 41,925 | 69,018 | 65,832 |
|  | Average length of genes (bp) | 11,959 | 8,834 | 8,192 |
|  | Average length of transcripts (nt) | 2,067 | 1,423 | 1,479 |
|  | Average length of ORF (bp)/genes with start & stop codon | 1,621/36,340 | 1,443/ 63,662 | 1,479/63,733 |
|  | Gene models supported by EST (%) | 77.2 | 67.5% | 62.5% |
| Exons | No. of exons per gene | 19.6 | 13.38 | 11.27 |
|  | Average length (bp) | 99.8 | 105 | 130 |
|  | Total length (Mb) | 82.1 | 98.2 | 97.3 |
| Introns | No. of genes with introns (%) | 95.3 | 83.4 | 80.3 |
|  | Average length (bp) | 499 | 561 | 622 |
|  | First two nucleotides at 5' splice sites | GT/GC/GA | GT/GC/GA | GT/GC/GA |
|  | Total length (Mb) | 331.5 | 481.8 | 421.2 |
| Intergenic regions | Average length (bp) | 2,064 | 2,008 | 2,202 |
|  | Unidirectional arrangement of genes | Yes | Yes | Yes |

*From Shoguchi et al. (2013)

**Table S4. Summary of assembled transcriptome contigs**

|  | *Symbiodinium* transcriptome assembly | | |
| --- | --- | --- | --- |
|  | SymB* | SymA | SymC |
| Total bases (megabases) | 65.83 | 83.59 | 72.59 |
| Number of unique sequences | 63,104 | 76,628 | 68,876 |
| Number of sequences mapped to the assembled scaffold (blastn, 1e–10) | 63,062 | 73,396 | 58,890 |
| GC contents (%) | 51.1 | 57.05 | 53.16 |
| Average length (nucleotides) | 1,043 | 1,090 | 1,054 |
| N50 size (nucleotides) | 1,684 | 1,774 | 1,686 |

*From Shoguchi et al. (2013)

**Table S5. Expanded genes having Pfam domains in SymA**

| Pfam ID | Pfam name | SymA | SymB | SymC | X-squared | Description from Pfam database |
| --- | --- | --- | --- | --- | --- | --- |
| PF00078.22 | RVT_1 | 888 | 310 | 816 | 295.5829 | Reverse transcriptase (RNA-dependent DNA polymerase) |
| PF13540.1 | RCC1_2 | 750 | 290 | 636 | 205.4368 | Regulator of chromosome condensation (RCC1) repeat |
| PF03372.18 | Exo_endo_phos | 404 | 112 | 177 | 203.4892 | Endonuclease/Exonuclease/phosphatase family |
| PF14529.1 | Exo_endo_phos_2 | 182 | 44 | 65 | 114 | Endonuclease-reverse transcriptase |
| PF02536.9 | mTERF | 74 | 1 | 10 | 111.8353 | Mitochondrial transcription termination factor |
| PF00023.25 | Ank | 1283 | 828 | 1046 | 98.4219 | Ankyrin repeat |
| PF13606.1 | Ank_3 | 1150 | 734 | 931 | 92.3005 | Ankyrin repeat |
| PF13637.1 | Ank_4 | 1225 | 796 | 996 | 91.6414 | Ankyrin repeats (many copies) |
| PF12796.2 | Ank_2 | 1324 | 885 | 1109 | 87.1374 | Ankyrin repeats (3 copies) |
| PF13857.1 | Ank_5 | 1228 | 823 | 1024 | 80.0137 | Ankyrin repeats (many copies) |
| PF00443.24 | UCH | 190 | 55 | 108 | 78.6346 | Ubiquitin carboxyl-terminal hydrolase |
| PF01885.11 | PTS_2-RNA | 152 | 34 | 142 | 78.3171 | RNA 2'-phosphotransferase, Tpt1 / KptA family |
| PF04665.7 | Pox_A32 | 39 | 0 | 0 | 78 | Poxvirus A32 protein |
| PF13884.1 | Peptidase_S74 | 40 | 5 | 3 | 54.125 | Chaperone of endosialidase |
| PF02338.14 | OTU | 107 | 31 | 50 | 49.9255 | OTU-like cysteine protease |
| PF00240.18 | ubiquitin | 218 | 137 | 107 | 42.8182 | Ubiquitin family |
| PF14448.1 | Nuc_N | 56 | 10 | 39 | 30.9143 | Nuclease N terminal |
| PF13812.1 | PPR_3 | 625 | 478 | 471 | 28.8272 | Pentatricopeptide repeat domain |
| PF13975.1 | gag-asp_proteas | 27 | 4 | 6 | 26.3243 | gag-polyprotein putative aspartyl protease |
| PF14528.1 | LAGLIDADG_3 | 23 | 3 | 4 | 25.4 | LAGLIDADG-like domain |
| PF00520.26 | Ion_trans | 703 | 616 | 528 | 24.8717 | Ion transport protein |
| PF00096.21 | zf-C2H2 | 49 | 11 | 29 | 24.3596 | Zinc finger, C2H2 type |
| PF00016.15 | RuBisCO_large | 24 | 4 | 5 | 23.0909 | Ribulose bisphosphate carboxylase large chain, catalytic domain |
| PF02429.10 | PCP | 34 | 8 | 11 | 22.9057 | Peridinin-chlorophyll A binding protein |
| PF03703.9 | bPH_2 | 13 | 1 | 0 | 22.4286 | Bacterial PH domain |
| PF00961.14 | LAGLIDADG_1 | 21 | 3 | 4 | 21.9286 | LAGLIDADG endonuclease |
| PF00504.16 | Chloroa_b-bind | 141 | 100 | 74 | 21.7333 | Chlorophyll A-B binding protein |
| PF13041.1 | PPR_2 | 734 | 610 | 582 | 20.3863 | PPR repeat family |
| PF13456.1 | RVT_3 | 35 | 6 | 22 | 20.0952 | Reverse transcriptase-like |
| PF04886.7 | PT | 22 | 1 | 11 | 19.4706 | PT repeat |

Domains of organelle proteins are highlighted in green.

Repeat domains are highlighted in gray.

**Table S6. Expanded genes having Pfam domains in SymC**

| Pfam ID | Pfam name | SymA | SymB | SymC | Chi-squared | Description from Pfam database |
| --- | --- | --- | --- | --- | --- | --- |
| PF07727.9 | RVT_2 | 1364 | 727 | 1599 | 330.9967 | Reverse transcriptase (RNA-dependent DNA polymerase) |
| PF00145.12 | DNA_methylase | 151 | 179 | 526 | 305.8621 | C-5 cytosine-specific DNA methylase |
| PF00665.21 | rve | 797 | 393 | 1057 | 298.9372 | Integrase core domain |
| PF00098.18 | zf-CCHC | 290 | 277 | 684 | 256.6379 | Zinc knuckle |
| PF00075.19 | RNase_H | 233 | 113 | 471 | 243.8286 | RNase H |
| PF13306.1 | LRR_5 | 79 | 233 | 355 | 172.078 | Leucine rich repeats (6 copies) |
| PF13917.1 | zf-CCHC_3 | 44 | 33 | 185 | 164.5267 | Zinc knuckle |
| PF05725.7 | FNIP | 82 | 112 | 281 | 145.3937 | FNIP Repeat |
| PF12688.2 | TPR_5 | 9 | 9 | 79 | 101.0309 | Tetratrico peptide repeat |
| PF00589.17 | Phage_integrase | 36 | 52 | 149 | 94.6582 | Phage integrase family |
| PF13374.1 | TPR_10 | 87 | 85 | 216 | 87.1289 | Tetratricopeptide repeat |
| PF13921.1 | Myb_DNA-bind_6 | 4 | 6 | 57 | 80.806 | Myb-like DNA-binding domain |
| PF13855.1 | LRR_8 | 111 | 264 | 279 | 79.2936 | Leucine rich repeat |
| PF00560.28 | LRR_1 | 68 | 105 | 193 | 67.5902 | Leucine Rich Repeat |
| PF12799.2 | LRR_4 | 158 | 217 | 310 | 51.4365 | Leucine Rich repeats (2 copies) |
| PF00692.14 | dUTPase | 2 | 1 | 31 | 51.2353 | dUTPase |
| PF13176.1 | TPR_7 | 118 | 123 | 228 | 49.3603 | Tetratricopeptide repeat |
| PF12895.2 | Apc3 | 71 | 92 | 167 | 46.3091 | Anaphase-promoting complex, cyclosome, subunit 3 |
| PF13504.1 | LRR_7 | 28 | 52 | 101 | 45.8895 | Leucine Rich Repeat (a dead family) |
| PF00642.19 | zf-CCCH | 202 | 110 | 229 | 43.1682 | Zinc finger C-x8-C-x5-C-x3-H type (and similar) |
| PF14216.1 | DUF4326 | 2 | 12 | 40 | 43.1111 | Domain of unknown function (DUF4326) |
| PF13976.1 | gag_pre-integrs | 8 | 8 | 43 | 41.5254 | GAG-pre-integrase domain |
| PF01391.13 | Collagen | 19 | 17 | 63 | 40.9697 | Collagen triple helix repeat (20 copies) |
| PF14787.1 | zf-CCHC_5 | 13 | 6 | 43 | 37.3871 | GAG-polyprotein viral zinc-finger |
| PF08483.6 | IstB_IS21_ATP | 0 | 0 | 18 | 36 | IstB-like ATP binding N-terminal |
| PF13432.1 | TPR_16 | 197 | 199 | 310 | 35.5439 | Tetratricopeptide repeat |
| PF14559.1 | TPR_19 | 137 | 140 | 233 | 35.0471 | Tetratricopeptide repeat |
| PF13424.1 | TPR_12 | 298 | 292 | 422 | 31.9289 | Tetratricopeptide repeat |
| PF13517.1 | VCBS | 21 | 22 | 62 | 31.2571 | Repeat domain in Vibrio, Colwellia, Bradyrhizobium and Shewanella |
| PF01839.18 | FG-GAP | 20 | 21 | 58 | 28.4242 | FG-GAP repeat |

Repeat domains are highlighted in gray.
