# Supplementary material for: Two divergent Symbiodinium genomes reveal conservation of a gene cluster for sunscreen biosynthesis and recently lost genes
Source: BMC Genomics. 2018 Jun 14;19:458. doi: 10.1186/s12864-018-4857-9 (PMC6001144; doi:10.1186/s12864-018-4857-9)
Supplement: Supplementary file 2 — Figure S1. A screen shot of scaffold 314 on the SymA genome browser, which is accessible via http://marinegenomics.oist.jp/gallery/, and RNAseq read counts showing the expressions of MAA biosynthetic genes. Figure S2. A molecular phylogenetic tree of O-methyltransferase. Figure S3. A molecular phylogenetic tree of ATP-grasp family proteins. Figure S4. A molecular phylogenetic tree of D-Ala D-Ala ligase family proteins. Figure S5. A molecular phylogenetic tree of cryptochromes/photolyases. (PDF 651 kb) [file 12864_2018_4857_MOESM2_ESM.pdf]

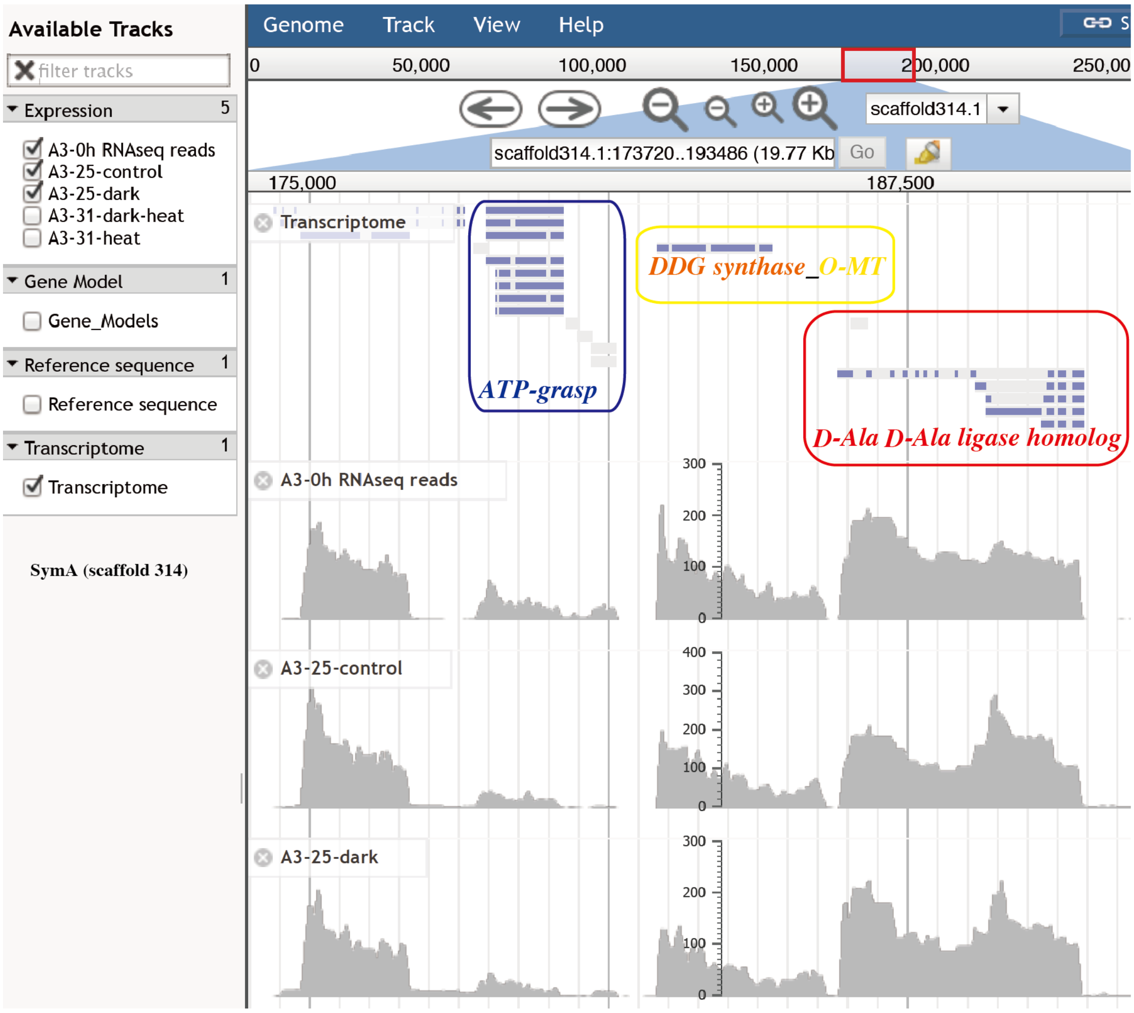

Fig. S1. A screen shot of scaffold 314 on the SymA genome browser, accessible at http://marinegenomics.oist.jp/gallery/ and RNAseq read counts showing expression of MAA biosynthetic genes

Transcriptome contigs for MAA biosynthetic genes, *ATP-grasp* (blue box), *DDG synthase_O-MT* (yellow box) and *D-Ala D-Ala ligase homolog* (red box), are displayed on the first column. RNAseq read counts for the genomic regions are shown in the second, third, and fourth columns, which correspond to mapped reads from each of the RNAseq libraries (Additional file 1: Table S2). The read counts show that expression levels between light (third column) and dark (fourth column) conditions were similar for the three genes.

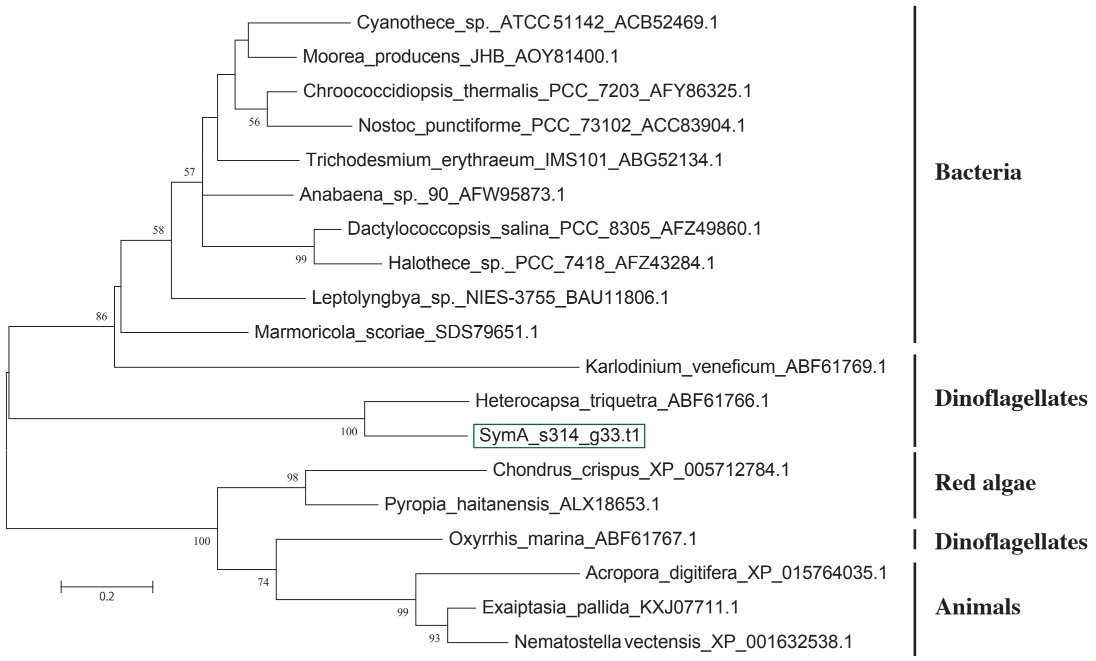

Figure S2. A molecular phylogenetic tree of *O*-methyltransferase

In this maximum likelihood tree, SymA *O*-methyltransferase (*O*-MT) forms a clade with that of the dinoflagellate, *Heterocapsa triquetra*. Accession numbers in NCBI and SymA gene model ID are followed by species names. The scale bar indicates the number of substitutions per site.

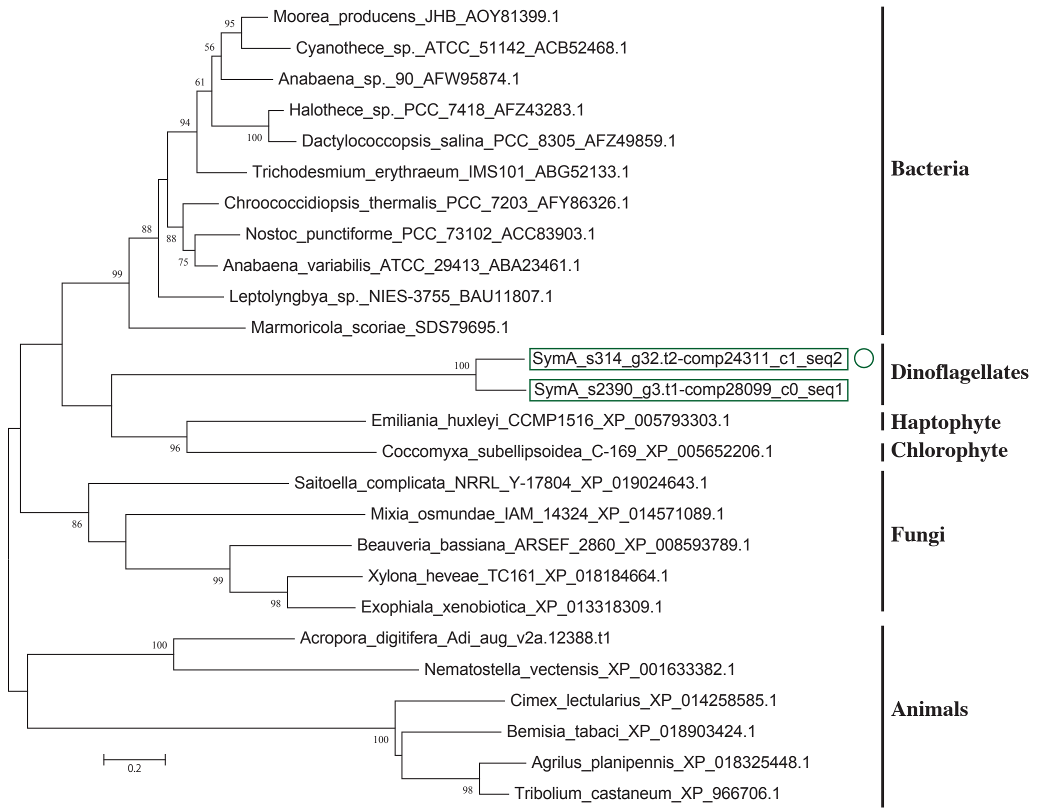

Figure S3. A molecular phylogenetic tree of ATP-grasp family proteins

In this maximum likelihood tree, two SymA ATP-grasp family proteins do not form a clade with those of bacteria. The SymA protein encoded in the MAA gene cluster is shown with a green circle. NCBI accession numbers and gene model-transcriptome contig IDs for SymA are followed by species names.

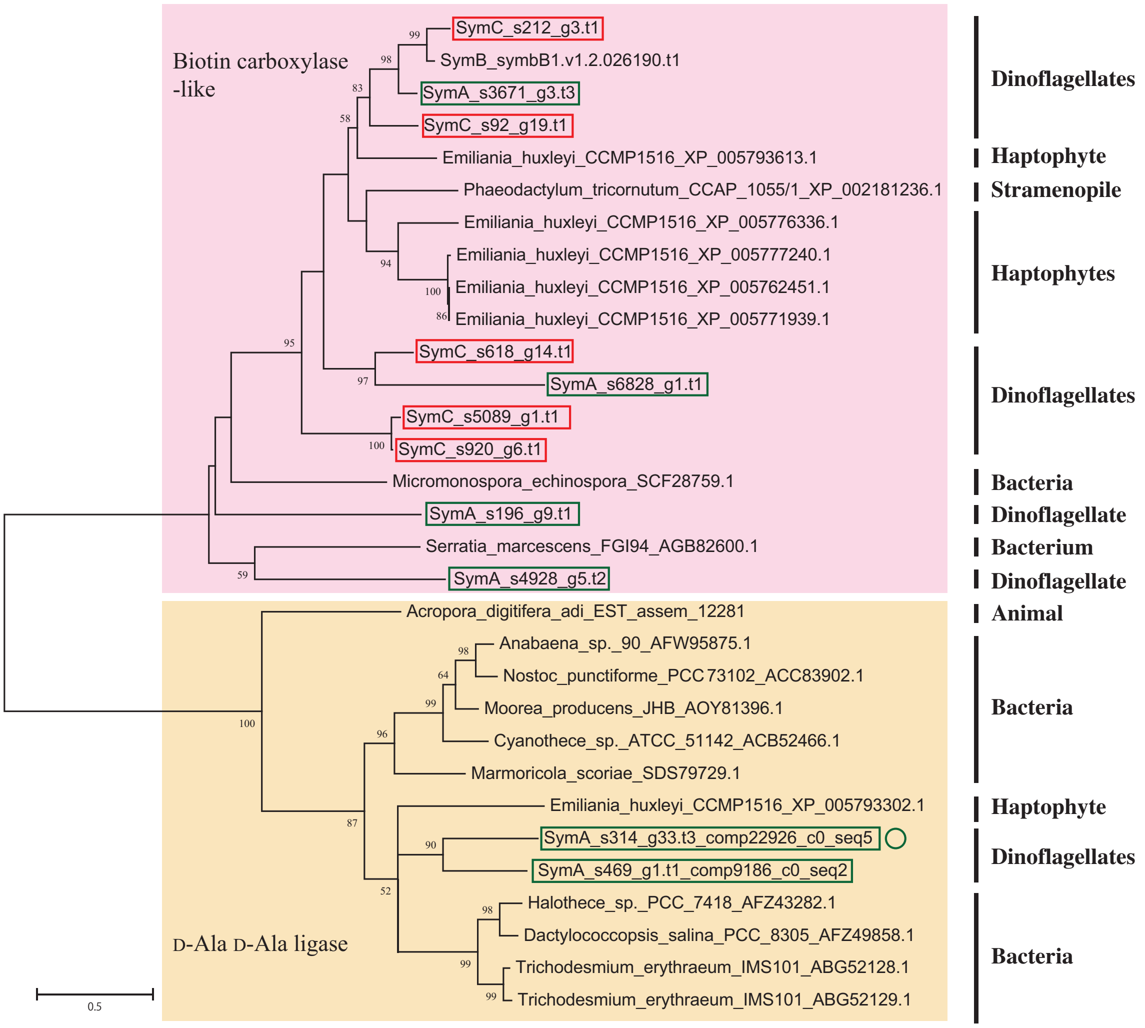

Figure S4. A molecular phylogenetic tree of D-Ala D-Ala ligase family proteins

In this maximum likelihood tree, two SymA D-Ala D-Ala ligases form a clade with those of bacteria and Haptophyceae. SymA protein, which is encoded in the MAA gene cluster, is shown with a green circle. Biotin carboxylases of bacteria are included as out-groups.

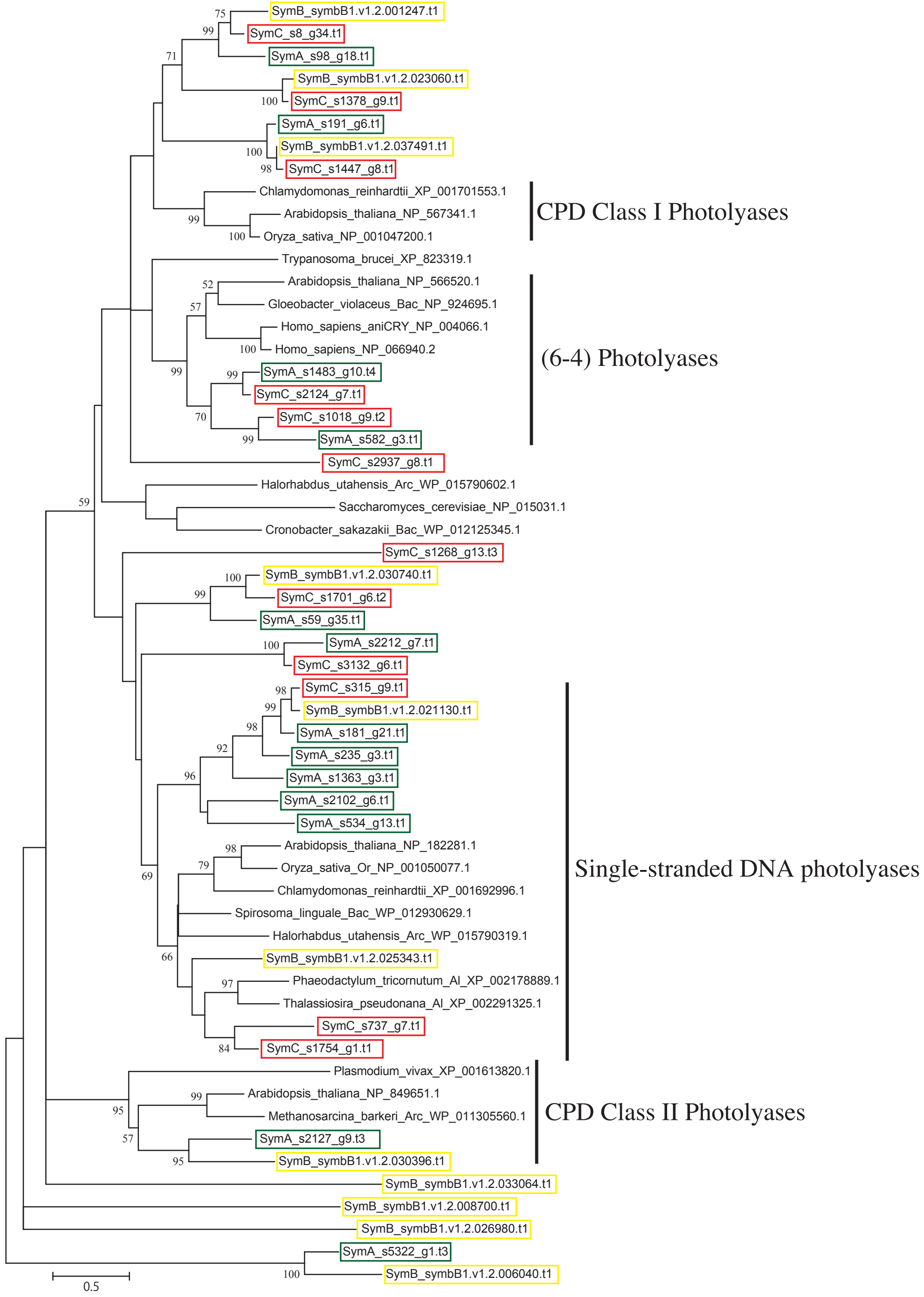

Figure S5. A molecular phylogenetic tree of cryptochromes/photolyases

This maximum likelihood tree of cryptochrome and photolyase genes indicates that gene numbers for potential DNA repair enzymes are not expanded in SymB (yellow) and SymC (red). NCBI accession numbers are followed by species names.
